# Surgeons’ Perspectives on Changing the Default Number of Doses for Opioid Prescriptions in Electronic Health Record Systems

**DOI:** 10.1001/jamanetworkopen.2023.15633

**Published:** 2023-05-26

**Authors:** Kao-Ping Chua, Marc C. Thorne, Chad M. Brummett, Melissa DeJonckheere

**Affiliations:** 1 Department of Pediatrics, Susan B. Meister Child Health Evaluation and Research Center, University of Michigan Medical School, Ann Arbor; 2 Department of Health Management and Policy, University of Michigan School of Public Health, Ann Arbor; 3 Division of Pediatric Otolaryngology, Department of Otolaryngology, University of Michigan Medical School, Ann Arbor; 4 Division of Pain Medicine, Department of Anesthesiology, University of Michigan Medical School, Ann Arbor; 5 Michigan Opioid Prescribing Engagement Network, University of Michigan Medical School, Ann Arbor; 6 Department of Family Medicine, University of Michigan Medical School, Ann Arbor

## Abstract

**Importance:**

An intervention in 2021 at a tertiary medical center found that the implementation of evidence-based default dosing settings for opioid prescriptions written in electronic health record systems was associated with reduced opioid prescribing to adolescents and young adults aged 12 to 25 years undergoing tonsillectomy. It is unclear whether surgeons were aware of this intervention, whether they thought the intervention was acceptable, or whether they believed similar interventions were feasible to implement in other surgical populations and institutions.

**Objective:**

To assess surgeons’ experiences and perspectives regarding an intervention that changed the default number of doses for opioid prescriptions to an evidence-based level.

**Design, Setting, and Participants:**

This qualitative study was conducted at a tertiary medical center during October 2021, 1 year after implementation of the intervention, in which the default number of doses for opioid prescriptions written through an electronic health record system to adolescents and young adults undergoing tonsillectomy was lowered to an evidence-based level. Semistructured interviews were conducted with otolaryngology attending and resident physicians who had cared for the adolescents and young adults undergoing tonsillectomy after implementation of the intervention. Factors that play a role in opioid prescribing decisions after surgery and participants’ awareness of and views on the intervention were assessed. The interviews were coded inductively and a thematic analysis was performed. Analyses were conducted from March to December 2022.

**Exposure:**

Change in the default dosing settings for opioid prescriptions written in an electronic health record system to adolescents and young adults undergoing tonsillectomy.

**Main Outcomes and Measures:**

Surgeons’ experiences and perspectives regarding the intervention.

**Results:**

The 16 otolaryngologists interviewed included 11 residents (68.8%), 5 attending physicians (31.2%), and 8 women (50.0%). No participant reported noticing the change in the default settings, including those who wrote opioid prescriptions with the new default number of opioid doses. From the interviews, 4 themes regarding surgeons' perceptions and experiences of the intervention emerged: (1) opioid prescribing decisions are influenced by patient, procedure, physician, and health system factors; (2) defaults may substantially influence prescribing behavior; (3) support for the default dosing setting intervention depended on whether it was evidence-based and had unintended consequences; and (4) changing the default dosing settings is potentially feasible in other surgical populations and institutions.

**Conclusions and Relevance:**

These findings suggest that interventions to change the default dosing settings for opioid prescriptions may be feasible to implement in a variety of surgical populations, particularly if the new settings are evidence-based and if unintended consequences are carefully monitored.

## Key Points

**Question:**

Did surgeons notice a change in the default dosing settings for discharge opioid prescriptions to adolescents and young adults undergoing tonsillectomy written through an electronic health record system, and what were their perspectives regarding this intervention?

**Findings:**

In this qualitative study that analyzed semistructured interviews of 16 otolaryngology attending and resident physicians, no participant reported noticing the new default dosing settings for discharge opioid prescriptions. In general, participants reported that changing default settings was a powerful and feasible way to decrease postoperative opioid prescribing in other surgical populations and institutions.

**Meaning:**

These findings can inform future efforts to change the default dosing settings for opioid prescriptions written after surgery.

## Teaser

This qualitative study assesses surgeons’ perspectives regarding a change in the default number of doses for opioid prescriptions written through an electronic health record system to adolescents and young adults undergoing tonsillectomy.

## Introduction

Surgeons write one-fifth of opioid prescriptions to adolescents and young adults, and tonsillectomy is a common indication for surgical opioid prescriptions in this age group.^1,2^ Consequently, rightsizing opioid prescriptions written for tonsillectomy could substantially decrease the number of leftover opioids available for misuse by adolescents and young adults.^3^ A promising approach to achieve this goal may be to implement evidence-based default settings for the number of doses in opioid prescriptions written through electronic health record (EHR) systems,^4,5,6,7,8,9,10,11,12^ the primary means by which controlled substances are prescribed in the US.^13^ This approach has several advantages, including low implementation costs and the preservation of clinician autonomy, the latter of which may increase clinician support.^14,15^

In our 2022 study,^14^ we collected data on opioid consumption from adolescents and young adults aged 12 to 25 years undergoing tonsillectomy on the pediatric otolaryngology service at Michigan Medicine and lowered the default number of opioid doses in the EHR system from 30 doses to 12 doses, a level that would meet the needs of most patients. Compared with a control group, the proportion of opioid prescriptions with 12 doses increased by 45.3 percentage points and the number of opioid doses prescribed decreased by 29.2%. Despite these changes, the intervention was not associated with changes in health care utilization for pain or patient-reported pain control.^14^

With regard to these findings, 2 key questions remain. First, it is unclear whether the otolaryngologists were aware of the intervention, which was deliberately not announced. Second, the otolaryngologists’ views regarding the acceptability and generalizability of this intervention are unknown. Closing these gaps could illustrate the mechanism of the association between changing default dosing settings and prescribing behaviors and inform the design of efforts to implement evidence-based default dosing settings in other surgical populations and institutions.

In this study, we conducted semistructured interviews with otolaryngology resident and attending physicians at Michigan Medicine to assess their experiences and perspectives regarding the default dosing setting intervention. To our knowledge, this is the first study to assess clinicians’ views on changing EHR defaults for opioid prescriptions in the surgical setting.

## Methods

This qualitative study was approved by the institutional review board of the University of Michigan Medical School. Participants were provided with an informed consent form via email, were assured that responses would remain confidential, and were compensated $150 for their time. This study followed the Consolidated Criteria for Reporting Qualitative Research (COREQ) reporting guideline for qualitative studies.

### Description of the Intervention

The intervention is described in detail in the 2022 study.^14^ In brief, we enrolled patients aged 12 to 25 years undergoing palatine tonsillectomy at Michigan Medicine from October 1, 2019, to July 31, 2021. The change, which was implemented on October 1, 2020, involved decreasing the default number of doses in a discharge order set used by the pediatric otolaryngology service from 30 doses to 12 doses. The control group included adolescents and young adults undergoing tonsillectomy on the general otolaryngology service who were not exposed to the intervention.

### Sample Recruitment

Eligible participants included attending physicians on the pediatric otolaryngology service and otolaryngology residents at Michigan Medicine. Residents complete 3-month rotations on the pediatric otolaryngology service during their 5-year residency. Pediatric otolaryngology attending physicians were considered eligible if they had cared for 1 or more adolescents or young adults undergoing tonsillectomy during the postintervention period (October 1, 2020, to July 31, 2021), and otolaryngology residents were considered eligible if they had written 1 or more discharge opioid prescriptions to an adolescent or young adult undergoing tonsillectomy during this period. Owing to the limited number of pediatric otolaryngology attending physicians, we did not require that eligible attending physicians write 1 or more discharge opioid prescriptions during the postintervention period. The first author (K.C.) invited all eligible physicians at the institution to participate via email.

### Interview Guide Development

A semistructured interview guide (eAppendix in Supplement 1) was developed by the research team and was informed by the domains in the Tailored Implementation in Chronic Diseases framework.^16^ This framework is widely used in implementation science to identify factors that could be associated with implementation of health care interventions. We first explored which factors modified participants’ opioid prescribing practices, both in general and specifically for adolescents and young adults undergoing tonsillectomy. We then explored whether participants had noticed the change in the default dosing settings, whether they supported or had concerns about this intervention, and whether they believe it was feasible to change the default dosing settings in other surgical populations and institutions.

### Interview Procedures

All interviews were conducted by the first author (K.C.), a male primary care pediatrician with doctoral training in health policy, expertise in opioid prescribing to adolescents and young adults,^1^ and prior experience in qualitative research.^17,18^ The first author led the 2022 study evaluating the default dosing intervention.^14^ Prior to the interview, participants were informed that the first author would be conducting the interview.

Interviews were conducted during October 2021, 3 months after the end of the study^14^ evaluating the association of the intervention with opioid prescribing and patient-reported outcomes. Interviews were conducted one-on-one using the video conferencing software Zoom version 5.13.11 (Zoom Video Communications). During interviews, participants were alone, either at work or at home. No repeated interviews were needed, and no field notes were constructed after the interview. Interviews lasted up to 60 minutes and were recorded and transcribed verbatim. Transcripts were not returned to participants for comment or correction, and participants did not provide feedback on findings.

### Statistical Analysis

Using a qualitative description approach,^19,20^ we conducted an inductive thematic analysis that allowed themes to emerge from the data.^21^ Interviews were coded by the first author (K.C.) and fourth author (M.D.), an expert in qualitative research.^18,22,23,24,25^ The 2 authors coded 3 transcripts independently. Disagreements were minor and were resolved via discussion using a consensus-based approach. This process repeated 3 to 4 transcripts at a time until all transcripts had been coded. After the final codebook was established, themes were developed by grouping related codes and describing participant experiences among the codes in each theme. Analyses were conducted using the software Excel version 2301 (Microsoft) and MAXQDA version 22 (VERBI Software). Analyses were conducted from March to December 2022.

## Results

### Sample

Of 20 eligible physicians at Michigan Medicine (7 attending physicians and 13 otolaryngology residents), 18 responded to the recruitment email. Only 2 individuals did not respond to the email; the reason for nonresponse was unknown. An additional 2 eligible participants remained willing to participate, but were unable to interview until November or December 2023. Because interviews had already revealed a rich variety of responses and began to consolidate around several main ideas, these 2 individuals were not included in the study. A total of 16 otolaryngologists were interviewed (11 residents [68.8%]; 5 attending physicians [31.2%]; 8 women [50.0%]). Among the 11 residents, 7 (63.6%) were in their first, second, or third year of residency. Among the 16 otolaryngologists, 10 (62.5%) had written 1 or more discharge opioid prescriptions with 12 doses to an adolescent or young adult undergoing tonsillectomy during the postintervention period. Of the 5 attending physicians, 3 had written 1 or more discharge opioid prescriptions with 12 doses. From the interviews, 4 main themes regarding surgeons' experiences and perceptions of the intervention emerged.

### Themes

#### Theme 1: Factors That Influence Opioid Prescribing Decisions

Surgeons reported that their opioid prescribing decisions are influenced by several factors (Table 1). These included patient factors (eg, age, comorbidities, personal or family history of opioid addiction, and patient and family expectations and preferences regarding opioids), procedure factors (eg, expected level of opioid need based on the invasiveness of the procedure or occurrence of complications), physician factors (eg, formative training on opioids and willingness to tolerate refills), and systems factors (eg, presence of a standardized care pathway, institutional or departmental culture, and policies such as insurance restrictions). In the context of tonsillectomy specifically, however, participants indicated that the influence of these factors was lessened and that opioids were almost always prescribed. One resident stated, “I think for tonsillectomy, I always offer [opioids]. It’s almost like the default is, I think you’re going to need a few doses of this medication and unless they say no in the preop setting, then I will prescribe them.”

**Table 1.** Codes and Example Quotations for Theme 1, Opioid Prescribing Decisions Are Influenced by Patient, Procedure, Physician, and Systems Factors

Subtheme and code	Example quotation	Physician role
Patient factors		
Age	“The older a patient gets with respect to tonsillectomy, I am certainly more willing to prescribe them [opioids] more knowing that just the general trend of course pain, postop pain gets much worse…”	Resident
Comorbidities other than opioid addiction	“If [the tonsillectomy] is for sleep apnea… I would tend to prescribe less for fear of depressing their respiratory system more.”	Resident
Patient or family history of opioid addiction	“I think that opioid abuse would certainly play a role in carefully how to discuss with them how many doses to prescribe.”	Attending
Patient and family preferences and expectations regarding opioids	“I would say the standard would be to prescribe an opioid. The reason I would not be if there was…after preoperative discussion, the family and patient really did not want to, then I would say I would respect their wishes after having a discussion of the risks and benefits…”	Attending
Expected level of opioid need based on history of pain or opioid use	“I think I will usually ask them if they’ve had surgery in the past and what they’ve taken in the past, depending on what type of surgery it is. If they are like, yes, I’ve always needed stuff, then I’ll tend to prescribe them something. If they say, no, I always do Tylenol and Motrin.”	Resident
Other medications the patient is taking	“…Patients who are on benzos or other pain medication, we would either avoid [opioids] or give, again, either a lower dose or fewer doses to avoid any conflict, any medication interactions.”	Resident
Procedure factors		
Expected level of opioid need based on type of surgery or occurrence of complications	“I think probably first and foremost, the type of procedure and anticipated pain level that is collected both through literature review, but also personal experience from performing that procedure in the past…”	Attending
Care setting	“One thing that also affects [opioid prescribing decisions] is if it’s going to be a planned admission or not. If they’re going straight home after a procedure, I’d prefer to try to get away without prescribing opioids. If they’re staying, then sometimes it’s nice to actually monitor how they do with pain, try a dose of opioids and monitor for side effects, because then it makes…it gives me a better idea of what will happen if I send them home with opioids.”	Resident
Risk of posttonsillectomy hemorrhage	“…there has been some suggestion that use of [non-steroidal anti-inflammatory medications] will increase post-tonsillectomy hemorrhage, and adults are more likely to have post-tonsillectomy hemorrhage, and so in that case we wouldn’t prescribe [ibuprofen]. So, they may need more oxycodone or some other narcotic in order to be able to get them through, since they don't have the second adjunct of ibuprofen.”	Resident
Physician factors		
Formative training on opioids	“The opioid crisis was something that was taught to us early on in training...I think a lot of my background and thoughts on [opioid prescribing] were gained in medical school.”	Resident
Willingness to tolerate refills	“I don’t know it’s important to avoid refills. I think it may be important to avoid unnecessary refills and so the question is, you know, are we giving the patients what they need?”	Attending
Desire to avoid refills	“I’d prefer not to have [patients] call in for a refill, partially because it just takes a really long time for them to get in via the phone service…if they’re after-hours calling [and] we have a million things to do, [we] often don’t get back to them for like 3, 4, or 5 hours because it’s not urgent…”	Resident
Attending and resident dynamics	“It really depends on the attending. Some of them…have pretty clear expectations that they either do or don’t want their patients to have opioids for a specific procedure, but I would say probably half the time it’s kind of left up to me to decide if they should get opioids and how many.”	Resident
Desire to avoid harms of opioids	“Well, I’ve just become really concerned about the risk of habit formation among patients and putting so many out into the community. So, I would say the vast majority of times that I have written for opioids, it’s for a very short course and I’d rather have patient need to call in, in order to get more pain medications than send them out with a lot.”	Resident
Systems factors		
Standardized pathway	“I think it’s good to have a standardized way because…I mean the-the ideal world is for all the residents to look up all the evidence for every single type of surgery and look up all the meta-analysis, all the, whatever, the publications online to really see what is the standard, but we don’t do that…that’s just a fact. So, I think it’s good that someone else in the department has done that work, whether it be a resident or some kind of taskforce and they come up with these so it’s easier for us to refer to that guideline and say, okay, this is what our department does.”	Resident
Institutional and departmental culture	“I think I think about [whether to prescribe opioids] much more consciously when I’m in the pediatric setting…I think if I didn’t prescribe an opioid for someone on the adult side who had tonsillectomy, the attending would think that I forgot or I wasn’t being an attentive resident…it’s definitely a default on the adult side.”	Resident
Insurance restrictions	“I think often adults will need opiates for seven to ten days and the insurance only allows five days at a time, sometimes only three. So, I’ll do five, tell them that’s the max they can do...”	Resident
Mandates to query the prescription drug monitoring program before prescribing opioids	“I believe there’s a stop [in the electronic health record] that if you prescribe over 3 to 5 days at a time you have to review [the state prescription drug monitoring program database] and things like that, which I do, but it’s just another, it’s just another double check that I…that it makes me think about it.”	Resident

Participants also indicated that opioid prescribing practices were highly standardized on the pediatric otolaryngology service, a major reason they usually prescribed the same number of doses for patients undergoing tonsillectomy on that service. In contrast, participants noted that there was less standardization on the general otolaryngology service, in which opioid prescribing decisions primarily reflected the attending physician’s preference.

#### Theme 2: Defaults’ Influence on Prescribing Behavior

The second theme is that defaults may substantially influence prescribing behavior*.* Table 2 includes example quotations for this theme. According to many participants, default dosing settings influenced their opioid prescribing behavior partly because they signal what is expected and appropriate. A resident stated, “I think it definitely makes it easier and probably makes us think, oh this…is the amount that should be given for this surgery. We’re very hardwired to believe Epic.”

**Table 2.** Codes and Example Quotations for Theme 2, Defaults May Substantially Influence Prescribing Behavior

Code	Example quotation	Physician role
Normative role of defaults	“It takes the decision out of your hands in factoring, oh, how many pills should I give them for this, or should I ask the attending how much they need.”	Resident
Impact of default varies based on experience and whether clinicians are attendings or residents	“I could see a couple colleagues be skeptical that [12 doses are] adequate for pain control after this procedure. Again, a lot of us trained with the teaching that this is an incredibly painful procedure, and you need traditionally required 50 plus or maybe even more…and so, there might be some resistance in people who were trained with that school of thought.”	Attending
Default settings leverage inertia	“…Just like in any other behavioral intervention, the defaults are sticky, so it takes a lot of inertia to overcome them.”	Resident
Dangers of defaults	“Sometimes [defaults are wrong]. Not speaking specifically to your order set, but sometimes it’ll default to like a very large number that is way more than we would ever give and sometimes it defaults to 1 dose.”	Resident
Trust in expert-developed opioid prescribing guidelines	“I think, like I think people have put a lot of effort into making the order set who are like way smarter than me too, and like thought about it and actually used data, so I just assume that it has thought behind it, and I’m pretty sure it does, like based on talking to other faculty members. So I feel like it’s a good starting off point.”	Resident
Belief that defaults changed opioid prescribing	“It obviously did change the way I prescribe, but not - not with my knowledge.”	Attending
Belief that defaults did not change opioid prescribing	“I don’t think so because I didn’t notice [the new defaults].”	Resident

However, some participants thought the normative power of defaults varies by clinical experience. When asked whether default dosing settings signaled appropriate prescribing behavior, one attending physician indicated, “I think it can, although not for me…but I think the residents, you know, can become so busy that they’re…heavily guided by order sets.”

In addition to the normative power of default dosing settings, some participants indicated that defaults also influence their prescribing behavior because they leverage inertia. A resident stated, “…I think anyone using the peds post-op order set is gonna click the default because it’s the default…we’re trying to go quickly between cases. We’re not gonna override things.”

No participants were aware that the default number of opioid doses had been decreased to 12 doses, including those who wrote 1 or more prescriptions with 12 doses during the postintervention period. Among these participants, several mistakenly believed that the new defaults had not changed the number of opioid doses they prescribed, a finding that perhaps supports the notion that defaults may substantially influence behavior.

#### Theme 3: Factors Affecting Support for the Intervention

The third theme is that support for changing the default dosing settings depended on whether it was evidence-based and had unintended consequences. Table 3 displays example quotations for this theme. Overall, most participants supported the intervention because they believed it could decrease the harms of opioids. For many participants, support for the intervention increased further when informed that the default number of doses was chosen on the basis of data on patient-reported opioid consumption. A resident noted, “It makes me more confident in prescribing that lower amount, knowing that it’s not just sort of a number that was just kind of pulled out of nowhere.”

**Table 3.** Codes and Example Quotations for Theme 3, Support for the Default Dosing Setting Intervention Depended on Whether It Was Evidence-Based and Had Unintended Consequences

Code	Example quotation	Physician role
Benefits of decreasing risk of opioid-related harms	“The benefit [of defaults] is clearly, you know, patients are taking less opiates at a vulnerable age where they potentially have the, you know, risk of becoming dependent or having extra that they may take, you know, in a recreational setting for teenagers.”	Resident
Importance of understanding the reason for defaults	“[I would be] curious to understand the rationale, but once I did, I certainly would be willing to adjust and update my practice.”	Attending
Concern that defaults could increase refills	“If refill requests were becoming so burdensome, it’s mostly to our nurses, that they were, it was adding to the workload to the point where we couldn’t answer other patient questions and was becoming really burdensome, then we might need to rethink [the defaults].”	Attending
Importance of evidence in shaping beliefs about defaults	“I mean it gives me a much more positive [reaction] to the change because this is a standard of practice that has evidence behind it.”	Attending

Some participants indicated that their support for efforts to change the default dosing settings depended on whether they understood the rationale for the change. One attending physician stated, “I generally try to understand the default in an order set because if you don’t know where it’s coming from, there’s a lot of different potentials, right? You know, was this an IT person in Wisconsin who said it, was it somebody with a lot of knowledge of usage, was it an outlier in your practice who wants to subversively change other peoples’ practice without them knowing? So as long as [there is a] reasonable rationale based on evidence…I’ll definitely go along the defaults.”

Several participants indicated that their support for the intervention would depend on whether it had unintended consequences, particularly an increase in refill requests. Some participants indicated that avoiding refills was particularly important in the context of tonsillectomy, because many community pharmacies do not compound liquid oxycodone, which is commonly prescribed after tonsillectomy owing to the difficulty of swallowing pills. However, many participants indicated that an increase in refill requests would not change their support of the intervention, as the additional burden would be outweighed by the potential reduction in opioid-related harms. Some participants even thought that refills were not an unintended consequence but rather an opportunity for clinicians to reassess pain control, including an attending physician who said, “I don’t think it would bother me to see an uptick in refills… I think that’s a prompt for, in my mind, you know, an opportunity to check in.”

#### Theme 4: Feasibility of Changing the Default Dosing Settings

The fourth theme to emerge from the interviews is that changing the default dosing settings is potentially feasible in other surgical populations and institutions. Table 4 displays example quotations for this theme. Most participants thought that it would be feasible to change the default dosing settings for opioid prescriptions written for a variety of surgical procedures, particularly if a discharge order set was already in place. An attending physician noted, “If there is already an order set available, it would be pretty easy just to change a number in that order set…For procedures where we don’t have a standard order set, which is the majority, it would take more work, because that would need to be created from scratch.”

**Table 4.** Codes and Example Quotations for Theme 4, Changing the Default Dosing Settings Is Potentially Feasible in Other Surgical Populations and Institutions

Code	Example quotation	Physician role
Defaults are feasible to implement for other procedures	“[Defaults] could be feasible as long as there is a procedure-based order set that all of the providers [clinicians] comply with using, and it includes an opioid in the order set. You know, up until recently, the opioid prescriptions have always been outside of the order set. It was everything besides the pain medications. But yes, it’s feasible if-if a division can get on board like we have with consistent order sets and the opioids included in that.”	Attending
Defaults are not feasible to implement for other procedures	“Tonsillectomy is the only surgery that I do where my postop orders always included opioid pain medication….I have an order set for an ear paper patch and a nasal cautery, but none of them require anything more than the nonopioid pain medications. For my practice, it would not be feasible to designate a default amount because I fill out those prescriptions as one-offs.”	Attending
Support for requiring justification for overriding defaults	“I probably would have been annoyed at first…but I think that it’s always nice to be reminded that you’re doing something that goes against the norm or goes against the expectation, I wouldn’t mind if there was a specific patient I wanted to give more to, I think that’s fine and it-it’s reasonable to expect me to at least stop and think about why I would be doing that.”	Resident
Opposition to requiring justification for overriding defaults	“[Electronic health record alerts are] annoying. I would have been annoyed. They slow us down.”	Resident

Other participants indicated that evidence on patient need for opioids for a procedure would further facilitate implementation. One attending physician stated, “Especially if you have an evidence base behind the number you chose, like you did, because then you’re much less likely to get negative, you know, blow back.”

Some participants thought that it would be more difficult to implement default dosing settings for procedures in which pain varies substantially between patients. Other participants indicated that implementation would be especially challenging if attending physicians at a given institution had strong, fixed preferences regarding the specific number of opioid doses that should be prescribed.

Participants were also asked whether it would be feasible in future efforts to require clinicians to provide justification when they override the default settings. On this point, views were mixed. Some participants were in support, including an attending physician who said, “Honestly, I think [requiring justification] would probably be pretty helpful because it would make me second guess, you know, or have to provide justification in my own mind why I’m deviating from the commonly accepted standard, and I wouldn’t be opposed to that.” Other participants thought requiring justification would be burdensome, including a resident who said, “I’d probably be annoyed…we could do 15 tonsillectomies in a day and so the amount of turnover between cases is tiny, it’s very, very fast so, anything that becomes burdensome quickly becomes annoying.” Some participants indicated that their support for requiring justification would depend on how frequently the alerts occurred, including an attending physician who stated, “It depends on how many times that would have to be overridden, you know. So if this something that you would do 5% to 10% of the time, then it wouldn’t be an annoyance. If it is something you felt that as an individual you had to do 80% to 90% of the time, then that would be an annoyance.”

## Discussion

This qualitative study explored otolaryngologists’ experiences and views regarding an intervention in which the default number of opioid doses in an EHR system was decreased. A key theme that emerged was the power of default settings to influence prescribing behavior. As an example of this power, none of the 16 participants was aware that the default number of doses had been decreased from 30 doses to 12 doses, yet 10 of the participants wrote 1 or more prescriptions with 12 doses after this change, and some of these participants mistakenly believed that they had not done so. These findings align with those of a 2019 study^10^ evaluating the impact of implementing a default setting of 15 doses for opioid prescriptions in the EHRs of 2 health systems. In that study, most prescribers interviewed were also unaware of this change and believed that the new default setting had not changed their behavior.^10^

Some participants in this study attributed the power of default dosing settings to the fact that it was easier to comply with these settings rather than take the time to override them. Others attributed this power to the normative nature of default dosing settings, indicating they trusted that the settings signaled the appropriate behavior. Of note, some participants thought that the normative power of default dosing settings would be greatest for inexperienced clinicians. In potential support of this notion, a 2018 study^9^ found that after implementation of a systemwide default number of doses for opioid prescriptions, surgical residents were the least likely to deviate from the new defaults. These considerations suggest that ensuring the appropriateness of default dosing settings may be an important goal for resident training, as inappropriate settings might create false perceptions of what constitutes appropriate care.

Participants identified several factors that might increase clinicians’ support for efforts to change the default dosing settings for opioid prescriptions. First, some thought that default settings should not automatically be trusted and that it was important to understand how the settings were developed. This finding suggests that transparency regarding the development of the default settings could increase clinicians’ support. Second, several participants were reassured when informed that the new default settings were based on patient-reported opioid consumption and were not arbitrarily set. This finding suggests that using evidence to set the default dosing settings could also increase clinicians’ support. Third, some participants expressed concerns that lowering the default number of doses in opioid prescriptions could increase refill requests. This finding suggests that carefully monitoring for changes in refills may be important to secure clinicians’ support. To further enhance support, those responsible for changing the default dosing settings could educate surgeons on the large body of literature showing that postoperative opioid prescribing can be decreased without increasing refills.^26,27,28^

This study revealed important directions for future research. For example, participants had mixed opinions on whether justification should be required for overriding a new default dosing setting for opioid prescriptions. Some thought this requirement would be a useful check on their decision-making. Others were concerned that the requirement would impede workflow, although 1 participant reported this concern might be mitigated if justification was only required some of the time. Further research is needed to evaluate whether requiring justification to override defaults affects clinician burden, opioid prescribing, and patient outcomes.

### Limitations

This study has limitations. First, the transferability of findings is unclear, as interviews were limited to 1 surgical population at a single institution. However, most participants were confident that evidence-based default dosing settings for opioid prescriptions could be implemented in other surgical populations and institutions. Second, we ideally would have only interviewed participants who were exposed both to the original and updated order set, but we were unable to do so owing to the limited number of otolaryngologists at our institution. However, nearly all eligible otolaryngologists participated, increasing confidence in the validity of the findings.

## Conclusions

In this qualitative study, most participants reported that implementing evidence-based default dosing settings would be a feasible approach to reduce opioid prescribing in a variety of surgical settings. Future studies should identify which strategies best facilitate implementation of such interventions.

## Notes

Supplement 1. **eAppendix.** Interview Guide

Supplement 2.
Data Sharing Statement

## References

[1] Chua KP, Brummett CM, Conti RM, Bohnert AS. Opioid prescribing to US children and young adults in 2019. Pediatrics. 2021;148(3):e2021051539. doi:10.1542/peds.2021-051539 34400571 PMC8778996

[2] Chua KP, Harbaugh CM, Brummett CM, . Association of perioperative opioid prescriptions with risk of complications after tonsillectomy in children. JAMA Otolaryngol Head Neck Surg. 2019;145(10):911-918. doi:10.1001/jamaoto.2019.2107 31393537 PMC6692671

[3] McCabe SE, West BT, Boyd CJ. Leftover prescription opioids and nonmedical use among high school seniors: a multi-cohort national study. J Adolesc Health. 2013;52(4):480-485. doi:10.1016/j.jadohealth.2012.08.007 23298996 PMC3608842

[4] Delgado MK, Shofer FS, Patel MS, . Association between electronic medical record implementation of default opioid prescription quantities and prescribing behavior in two emergency departments. J Gen Intern Med. 2018;33(4):409-411. doi:10.1007/s11606-017-4286-5 29340937 PMC5880773

[5] Bursua A, Mudreac A, Koppen L, Larson C, Park YS, Sreedhar R. Effect of default order standardization on opioid prescribing patterns. Jt Comm J Qual Patient Saf. 2021;47(7):431-437. doi:10.1016/j.jcjq.2021.03.005 33896745

[6] Slovis BH, Riggio JM, Girondo M, . Reduction in hospital system opioid prescribing for acute pain through default prescription preference settings: pre-post study. J Med Internet Res. 2021;23(4):e24360. doi:10.2196/24360 33851922 PMC8082388

[7] Carlson A, Nelson ME, Patel H. Longitudinal impact of a pre-populated default quantity on emergency department opioid prescriptions. J Am Coll Emerg Physicians Open. 2020;2(1):e12337. doi:10.1002/emp2.12337 33521788 PMC7819264

[8] Montoy JCC, Coralic Z, Herring AA, Clattenburg EJ, Raven MC. Association of default electronic medical record settings with health care professional patterns of opioid prescribing in emergency departments: a randomized quality improvement study. JAMA Intern Med. 2020;180(4):487-493. doi:10.1001/jamainternmed.2019.6544 31961377 PMC6990860

[9] Chiu AS, Jean RA, Hoag JR, Freedman-Weiss M, Healy JM, Pei KY. Association of lowering default pill counts in electronic medical record systems with postoperative opioid prescribing. JAMA Surg. 2018;153(11):1012-1019. doi:10.1001/jamasurg.2018.2083 30027289 PMC6583068

[10] Zivin K, White JO, Chao S, . Implementing electronic health record default settings to reduce opioid overprescribing: a pilot study. Pain Med. 2019;20(1):103-112. doi:10.1093/pm/pnx304 29325160

[11] Meisenberg BR, Grover J, Campbell C, Korpon D. Assessment of opioid prescribing practices before and after implementation of a health system intervention to reduce opioid overprescribing. JAMA Netw Open. 2018;1(5):e182908. doi:10.1001/jamanetworkopen.2018.2908 30646184 PMC6324493

[12] Bachhuber MA, Nash D, Southern WN, . Effect of changing electronic health record opioid analgesic dispense quantity defaults on the quantity prescribed: a cluster randomized clinical trial. JAMA Netw Open. 2021;4(4):e217481. doi:10.1001/jamanetworkopen.2021.7481 33885773 PMC8063068

[13] Surescripts. National Progress Report 2020. March 2021. Accessed August 19, 2021. https://surescripts.com/news-center/national-progress-report-2020

[14] Chua KP, Thorne MC, Ng S, Donahue M, Brummett CM. Association between default number of opioid doses in electronic health record systems and opioid prescribing to adolescents and young adults undergoing tonsillectomy. JAMA Netw Open. 2022;5(6):e2219701. doi:10.1001/jamanetworkopen.2022.19701 35771572 PMC9247741

[15] Delgado MK. Patient-centered default opioid orders-a path forward for postoperative opioid stewardship. JAMA Netw Open. 2022;5(6):e2219712. doi:10.1001/jamanetworkopen.2022.19712 35771581

[16] Flottorp SA, Oxman AD, Krause J, . A checklist for identifying determinants of practice: a systematic review and synthesis of frameworks and taxonomies of factors that prevent or enable improvements in healthcare professional practice. Implement Sci. 2013;8:35. doi:10.1186/1748-5908-8-35 23522377 PMC3617095

[17] Schuiteman S, Chua KP, Plegue MA, Ilyas O, Chang T. Self-management of health care among youth: implications for policies on transitions of care. J Adolesc Health. 2020;66(5):616-622. doi:10.1016/j.jadohealth.2020.01.009 32113903 PMC7980769

[18] DeJonckheere M, Waselewski M, Amaro X, Frank A, Chua KP. Views on COVID-19 and use of face coverings among U.S. youth. J Adolesc Health. 2021;68(5):873-881. doi:10.1016/j.jadohealth.2021.02.015 33896552 PMC8061118

[19] Bradshaw C, Atkinson S, Doody O. Employing a qualitative description approach in health care research. Glob Qual Nurs Res. 2017;4:2333393617742282. doi:10.1177/2333393617742282 29204457 PMC5703087

[20] Thorne S, Kirkham SR, MacDonald-Emes J. Interpretive description: a noncategorical qualitative alternative for developing nursing knowledge. Res Nurs Health. 1997;20(2):169-177. doi:10.1002/(SICI)1098-240X(199704)20:2<169::AID-NUR9>3.0.CO;2-I 9100747

[21] Braun V, Clarke V. Using thematic analysis in psychology. Qual Res Psychol. 2006;3(2):77-101. doi:10.1191/1478088706qp063oa

[22] DeJonckheere M, McKee MM, Guetterman TC, . Implementation of a hearing loss screening intervention in primary care. Ann Fam Med. 2021;19(5):388-395. doi:10.1370/afm.2695 34546945 PMC8437567

[23] DeJonckheere M, Joiner KL, Ash GI, . Youth and parent perspectives on the acceptability of a group physical activity and coping intervention for adolescents with type 1 diabetes. Sci Diabetes Self Manag Care. 2021;47(5):367-381. doi:10.1177/26350106211040429 34610760

[24] Harbaugh CM, Vu JV, DeJonckheere M, Kim N, Nichols LP, Chang T. Youth perspectives of prescription pain medication in the opioid crisis. J Pediatr. 2020;221:159-164. doi:10.1016/j.jpeds.2020.02.003 32143929

[25] DeJonckheere M, Nichols LP, Moniz MH, . MyVoice national text message survey of youth aged 14 to 24 years: study protocol. JMIR Res Protoc. 2017;6(12):e247. doi:10.2196/resprot.8502 29229587 PMC5742661

[26] Howard R, Waljee J, Brummett C, Englesbe M, Lee J. Reduction in opioid prescribing through evidence-based prescribing guidelines. JAMA Surg. 2018;153(3):285-287. doi:10.1001/jamasurg.2017.4436 29214318 PMC5885924

[27] Vu JV, Howard RA, Gunaseelan V, Brummett CM, Waljee JF, Englesbe MJ. Statewide implementation of postoperative opioid prescribing guidelines. N Engl J Med. 2019;381(7):680-682. doi:10.1056/NEJMc1905045 31412184 PMC7160762

[28] Howard R, Fry B, Gunaseelan V, . Association of opioid prescribing with opioid consumption after surgery in Michigan. JAMA Surg. 2019;154(1):e184234. doi:10.1001/jamasurg.2018.4234 30422239 PMC6439853

