# Supplementary material for: Surgeons’ Perspectives on Changing the Default Number of Doses for Opioid Prescriptions in Electronic Health Record Systems
Source: JAMA Netw Open. 2023 May 26;6(5):e2315633. doi: 10.1001/jamanetworkopen.2023.15633 (PMC10220515; doi:10.1001/jamanetworkopen.2023.15633)
Supplement: Supplement 1. — eAppendix. Interview Guide [file jamanetwopen-e2315633-s001.pdf]

Supplemental Online Content

Chua KP, Thorne MC, Brummett CM, DeJonckheere M. Surgeons' perspectives on changing the default number of doses for opioid prescriptions in electronic health record systems. *JAMA Netw Open*. 2023;6(5):e2315633. doi:10.1001/jamanetworkopen.2023.15633

eAppendix. Interview Guide

This supplemental material has been provided by the authors to give readers additional information about their work.

© 2023 Chua KP et al. *JAMA Network Open.*

eAppendix. Interview Guide.

Thank you so much for taking the time to speak with us today. We are interested in learning more about opioid prescribing to adolescents and young adults aged 12-25 years who undergo tonsillectomy with or without adenoidectomy.

Before we proceed, I would like to review the informed consent form that we sent you. We expect this interview to take no more than 45 minutes. Participating in interviews is voluntary. You can skip any questions that you don't want to answer, whatever the reason, and you don't have to tell us why.

This interview will be audio-recorded to ensure that we record your views accurately. The results of this study, including direct quotations, could be published in an article, but your identity will be kept confidential. The quotations will not be attributed to you in a way that would allow others to identify who you are. We will take every precaution necessary and use strict security measures to protect your data and privacy. Your name will be replaced with a random code to hide your identity while the data is stored. Under no circumstances will we intentionally release your name or other identifying information publicly. As a rule, the researchers will continue to use information about you until the study is over and will keep it secure until it is destroyed.

Do you have any questions? [Answer any questions]

Do we have your verbal consent to proceed with the interview? [If yes, then proceed; if no, then stop interview here and thank them]. [START RECORDING]

Warm-up questions

To begin with, we'd like to know a little more about you.

- [For residents] Could you describe which rotations you have done so far in residency?
- [For attendings] Could you describe what types of patients you see in your practice?

General views on opioid prescribing after surgery

Thanks for that background. We'd now like to explore your views on opioid prescribing after surgery in general, regardless of patient age or the type of procedure.

- What factors influence whether you prescribe an opioid after surgery?
- What factors influence how many opioid doses you prescribe after surgery?
- How comfortable are you in your ability to appropriately prescribe opioids?
- What is your view of the role of opioid prescriptions after surgery in the U.S. opioid epidemic?
-
- What is your view on opioid prescribing guidelines for surgery?

Views on opioid to adolescents and young adults for tonsillectomy

We’d now like to explore your views on opioid prescribing specifically among adolescents aged 12-17 years and young adults aged 18-25 years undergoing tonsillectomy.

- Tell me about the last time you prescribed an opioid to an adolescent or young adult undergoing tonsillectomy
- Walk me through the process of how you would write an opioid prescription to an adolescent or young adult undergoing tonsillectomy at the University of Michigan.
- What influences whether you prescribe an opioid to adolescents and young adults after tonsillectomy?
- What influences how many doses of opioids you prescribe to adolescents and young adults after tonsillectomy?
- How does your decision-making differ when prescribing opioids to adolescents aged 12-17 years versus young adults aged 18-25 years, if at all?
- How does your decision-making differ when prescribing opioids to adolescents aged 12-17 years versus young children < 12 years, if at all?

<u>Specific probes</u>

- In your view, how many doses is appropriate to prescribe for adolescents and young adults undergoing tonsillectomy?
- When considering how many doses to provide in an opioid prescription to an adolescent or young adult undergoing tonsillectomy, what role does patient satisfaction play in your thinking?
- When considering how many doses to provide in an opioid prescription to an adolescent or young adult undergoing tonsillectomy, what role does avoiding refills play in your thinking?
- When considering how many doses to provide in an opioid prescription to an adolescent or young adult undergoing tonsillectomy, what role do patient co-morbidities play in your thinking?
- When considering how many doses to provide in an opioid prescription to an adolescent or young adult undergoing tonsillectomy, what role do the preferences of other staff, such as PACU nurses, nurse practitioners, and physician assistants, play in your thinking?
- [For residents only] How does your attending influence your opioid prescribing practices to adolescents and young adults after tonsillectomy, if at all?
- [For attendings only] How do you convey your preferences to residents regarding opioid prescribing to adolescents and young adults after tonsillectomy, if at all?
- How do opioid prescribing practices to adolescents and young adults undergoing tonsillectomy vary between the pediatric and general otolaryngology service at the University of Michigan?

Views on intervention (change in default number of doses for opioid prescriptions)

© 2023 Chua KP et al. *JAMA Network Open.*

Now we'd like to ask you about any changes to your opioid prescribing practices to adolescents and young adults undergoing tonsillectomy over the last year.

- Over the past year, have your opioid prescribing practices to adolescents and young adults undergoing tonsillectomy changed? If yes, how so?

Specific probes

- Over the past year, did you notice a change to the default number of doses in opioid prescriptions to adolescents and young adults undergoing tonsillectomy written in MiChart?

  [If NO then say: In October 2020, we revised the tonsillectomy orderset at Mott Hospital so that the default number of doses for adolescents and young adults decreased from 30 to 12 on the pediatric service only.]

Views on the intervention

- How do you feel about this change?
- In your view, what might be the benefits and harms of prescribing 12 doses rather than a higher amount?
- What would make you more comfortable with prescribing 12 doses, if anything?
- The default number of doses was based on patient-reported opioid consumption data. Does that change your view about the intervention?
- How does 12 doses compare with what you normally prescribe to an adolescent or young adult undergoing tonsillectomy?
- Does the default number of doses in MiChart signal to you what number of doses is appropriate to prescribe?

Changes in response to the intervention

- Did the change in the default number of doses change your opioid prescribing practices? Why or why not? Do you have any specific examples?
- What issues, if any, have arisen from the change in the default number of doses from 30 to 12? Do you have any specific examples?
- [For residents] Did the new default dosing settings affect how you prescribed to patients undergoing tonsillectomy on the general otolaryngology service?
- [For those who prescribed 12 doses at least once] How did you feel about prescribing 12 doses the first time you did so?
- [For those who prescribed 12 doses at least once] Did patients raise any concerns when you prescribed 12 doses?

© 2023 Chua KP et al. *JAMA Network Open.*

Factors that influence response to the default dosing intervention

- In general, what factors might prompt a UM otolaryngology attending or resident to prescribe a different number of doses than the default of 12?
- Would it have affected your prescribing behavior if the chief of pediatric otolaryngology had announced that the new division standard was to write for 12 doses of opioids for adolescents and young adult after tonsillectomy?
- How did the culture of opioid prescribing on the pediatric otolaryngology service affect your decisions on whether to stay with the new default dosing settings?
- In your view, which people involved with the care of adolescents and young adults undergoing tonsillectomy would support the new default number of doses and why?
- In your view, which people involved with the care of adolescents and young adults undergoing tonsillectomy would oppose the new default number of doses and why?

Questions about the design of the intervention

- If we had made the default number of doses harder to override by requiring justification, would that have affected your prescribing behavior?
- Was it confusing to have different options in the orderset depending on whether the patient weighed less than 50 kg or more than 50 kg?
- How difficult was it to override the default number of doses and prescribe a different number?

Questions about scalability of the intervention

- Would it be feasible to change the default dosing settings for other surgical procedures?
- [For residents] How would changing the default dosing settings for patients undergoing tonsillectomy on the general otolaryngology service affect your prescribing?

Wrap-up

Is there anything else you'd like to add that we haven't covered?

Thank you for your time!

© 2023 Chua KP et al. *JAMA Network Open.*
