# Supplementary material for: Surgeons’ Perspectives on Changing the Default Number of Doses for Opioid Prescriptions in Electronic Health Record Systems
Source: JAMA Netw Open. 2023 May 26;6(5):e2315633. doi: 10.1001/jamanetworkopen.2023.15633 (PMC10220515; doi:10.1001/jamanetworkopen.2023.15633)
Supplement: Supplement 2. — Data Sharing Statement [file jamanetwopen-e2315633-s002.pdf]

Data Sharing Statement

Chua. Surgeons' Perspectives on Changing the Default Number of Doses for Opioid Prescriptions in Electronic Health Record Systems. *JAMA Netw Open*. Published May 26, 2023. doi:10.1001/jamanetworkopen.2023.15633

Data
Data available: No
